# Driving innovation from discovery to access: Meeting report of the 7 ^th^ Global Forum on TB Vaccines (8-10 October 2024, Rio de Janeiro, Brazil)

**DOI:** 10.12688/gatesopenres.16360.1

**Published:** 2025-08-27

**Authors:** Shaun Palmer, Rebecca A. Clark, Bridgette J. Connell, Vanessa Mwebaza Muwanga, Arthur Coelho, Paul Ogongo, Carly Young

**Affiliations:** 1 Stichting International Aids Vaccine Initiative, Amsterdam, 1013 NH, The Netherlands; 2 Stop TB Partnership Working Group on New Vaccines, New York, NY 10004, USA; 3 London School of Hygiene and Tropical Medicine, London, WC1E 7HT, UK; 4 University of Cape Town Department of Pathology, Cape Town, Western Cape, 7925, South Africa; 5 Universidade Federal de Goias Instituto de Patologia Tropical e Saude Publica, Goiânia, State of Goiás, 8QF3+XW, Brazil; 6 University of California San Francisco Department of Medicine, San Francisco, California, 94143, USA

**Keywords:** Tuberculosis, Vaccine, Clinical trial, Access, Funding, Community, Advocacy

## Abstract

We urgently need novel, effective, and accessible vaccines to end tuberculosis (TB) as a public health crisis. The 7th Global Forum on TB Vaccines was convened from 8–10 October 2024 in Rio de Janeiro, Brazil. Under the theme of “Driving innovation from discovery to access,” the program covered the breadth of TB vaccine research and development (R&D) through implementation, while underscoring the need for greater innovation and investments to advance development and ensure rapid, affordable, and equitable access. Participants shared the latest research on: approaches to diversify the TB vaccine pipeline, candidates advancing through late-stage trials toward licensure, and efforts to ensure new TB vaccines reach the populations that most need them. The forum provided a platform to learn from diverse experts across the field, including researchers, industry, funders, civil society, and affected communities. Participants examined cross-cutting enablers throughout, including opportunities to establish novel partnership and financing models, enhance open science, optimize R&D practices, and strengthen leadership and engagement with community members and high burden countries alike. In this report, we synthesize key themes and findings from the meeting, highlighting progress and priorities in the TB vaccine field.

## 1. Introduction

The Global Forum on Tuberculosis (TB) Vaccines is the world’s largest gathering of stakeholders interested in the development and deployment of new vaccines to prevent TB. The 7th Global Forum on TB Vaccines was convened from 8–10 October 2024 in Rio de Janeiro, Brazil—the first time this important conference had been held in the Americas—with over 300 participants in attendance. Under an overarching theme of “ *Driving innovation from discovery to access*,” the program covered the full spectrum of TB vaccine research and development (R&D), from basic and discovery research to diversifying the TB vaccine pipeline and clinical trials. With multiple candidates advancing through late-stage trials, delegates also explored strategies to ensure new TB vaccines reach the populations that most need them as quickly as possible to maximize public health impact. This focus on end-to-end development provided a unique opportunity for participants to hear diverse perspectives and learn and engage with experts from across the field, including insights from related advocacy, policy, and resource mobilization initiatives.

**Ethel Maciel** (Secretary of Health Surveillance & Environmental Health, Ministry of Health, Brazil) and **David Lewinsohn** (Chair, Stop TB Partnership Working Group on New TB Vaccines, USA) welcomed participants in the opening session with reflections on the diversity of approaches being explored in TB vaccine R&D and implementation, underpinned by global, multi-disciplinary collaboration in support of a clinical pipeline of at least 15 vaccine candidates.

The keynote address was delivered by **Jeremy Farrar** (Chief Scientist, World Health Organization (WHO), Switzerland) who challenged the WHO and global community to anticipate progress and prepare for success—not merely react to it, which inevitably contributes to inequity by delaying access. To do so, it is critical to urgently harness the expertise of diverse partners and prepare for the regulatory and logistical challenges of deploying future TB vaccines, including greater innovation for bridging studies and regulatory approvals for innovative trial design. In this regard, Farrar reflected on the work of the WHO TB Vaccine Accelerator Council, launched in 2023, to expedite and convene partners from around the world to accelerate equitable access to new TB vaccines ^1^; a financing and access working group is set to launch in 2025.

A series of high-level remarks during the opening and closing sessions explored the potential for innovation across the TB vaccine development to implementation continuum. **Michel Kazatchkine** (Switzerland) underscored the need for bold multilateral and multidisciplinary collaboration, noting that no country alone can end TB and emphasizing that this must be done in alignment with the principles of open and transparent science. **Sania Nishtar** (CEO, Gavi, Switzerland) highlighted the need for global stakeholders to prepare now for new TB vaccines, including developing innovative financing and procurement approaches to ensure equitable access, particularly in high burden countries (HBCs), at a time of stagnating international funding. **Mark Feinberg** (President & CEO, IAVI, USA) stressed the importance of sustainable TB vaccine R&D, including long-term global investments to fund efficacy trials and maintain a diverse pipeline, as late-stage candidates could yet fail. **Nina Russell** (Director, TB & HIV R&D, Gates Foundation, USA) called for innovative partnership and funding models for late-stage clinical development in global health more broadly, such as an aggregator fund with diverse funders for which TB could be a key target. Russell also called for greater investment in basic science to resolve pressing challenges, such as the identification of correlates of protection (CoP) and markers for vaccine efficacy. In a similar vein, **Lucica Ditiu** (Executive Director, Stop TB Partnership, Switzerland) called for more people to advocate and ask for concrete commitments and funding for TB vaccine development, including pursuing unconventional or as yet unexplored funding sources. **Miguel Aragón** (Coordinator of the Communicable Diseases & Environmental Determinants of Health Unit, Pan American Health Organisation (PAHO)/WHO, Brazil) emphasized that financing vaccine development is an intelligent investment before reflecting on regional commitments in South America, including Brazil’s role as co-chair of the TB Vaccine Accelerator Council. **Ricardo Alexandre Arcêncio** (President, REDE-TB, Brazil) called for sustained political pressure to fulfill commitments endorsed in the political declaration of the 2023 United Nations High-Level Meeting on TB (UN HLM on TB), including to invest US$5 billion annually in TB research by 2027 and develop and deliver a new TB vaccine within five years. ^2^

**Ruben Rizzi** (Senior Vice President, Global Regulatory Affairs, BioNTech, Germany) discussed translating insights from the COVID-19 response into a long-term framework for vaccine development, including leveraging platforms like mRNA that offer flexibility to adapt rapidly. **Ole Olesen** (Executive Director, TuBerculosis Vaccine Initiative, Netherlands) highlighted the need to attract and retain researchers, including early career researchers, to sustain the field, while underscoring the need for inclusive participation of researchers from HBCs. **Mario Moreira** (President, Fiocruz, Brazil) raised three key considerations to expand local manufacturing capacity to meet future demand: (1) plan for scale-up during late-stage development; (2) conduct demand forecasting in collaboration with governments; and (3) invest in novel technologies and partnerships. **Renato Cony Seorida** (Undersecretary for Health Promotion, Primary Care & Surveillance, Secretary of Health, Rio de Janeiro, Brazil) drew attention to the need to strengthen local primary healthcare and surveillance capacities to provide the highest standard of TB care, including for TB vaccine delivery. **Suvanand Sahu** (Deputy Executive Director, Stop TB Partnership, Switzerland) called for affected communities to be involved in the full R&D to implementation continuum to ensure meaningful impact. **Paulina Siniatkina** (artist and TB survivor, Netherlands) situated the efforts of the field in the context of affected communities by sharing the demands for inclusive and equitable development and implementation outlined in the Community Declaration, which was endorsed by over 1,400 individuals and organizations around the world. ^3^

## 2. Diversifying the TB vaccine pipeline

Diversifying the pipeline of TB vaccine candidates through innovative approaches in basic, translational and preclinical research is a priority for the field. A range of promising approaches were covered throughout the Forum on advancing early-stage research and a robust preclinical pipeline, exploring diverse immunologic approaches; improving formulations, vaccine platforms, and delivery routes; and optimizing animal models.

### 2.1 Broaden antigen targets and employ novel adjuvants

Novel antigen targets are needed to expand the limited repertoire currently under investigation. **Ely Van Riet** (TuBerculosis Vaccine Initiative, Netherlands) reflected on how not all vaccines will work equally well, noting that development of TB vaccines is challenging due to the proficient immune escape mechanisms of *Mtb.* Researchers must thus ask relevant questions when designing vaccine candidates. For example, when selecting antigenic targets it is important to understand if antigens are TB stage specific and whether they may reduce vaccine efficacy if involved in the immune escape mechanism of *Mtb.* **Paul Ogongo** (University of California San Francisco, USA) presented one approach demonstrating that rare variable *Mtb* antigens (RVMA) under diversifying evolutionary selection preferentially promote human Th17 responses ^4^ previously shown to be enriched in *Mtb* infected human lungs ^5^ and are associated with preventing progression to TB disease, ^6, 7^ positioning these antigens as potential candidates for prevention of disease (PoD) vaccines. In complementary studies, **Zach Howard** (University of California San Francisco, USA) shared promising results on a subset of RVMA delivered as a DNA vaccine cassette in mice which induced protective lung CD4 T cell responses, and reduced lung necrosis and inflammation (unpublished data). Additional research is planned to further investigate mechanisms of protection and the association with a Th17-polarizing response. Additionally, **Lyudmila Kovalchuke** (VIB-UGent, Belgium) presented the BAXERNA project ( www.baxerna.eu), which uses immunopeptidomics as a mass spectrometry-based approach to antigen discovery, ^8, 9^ coupled with novel mRNA vaccine formulations. ^10^ BAXERNA has identified over 200 *Mtb* immunopeptides from 175 different proteins, 16 of which are planned to undergo preclinical evaluation in mRNA vaccines. The top-performing antigens are then planned to be tested in a Phase 1 clinical trial.

The role of adjuvants in enhancing vaccine responses was demonstrated by **Lazaro Moreira Marques-Neto** (Instituto Butantan, Brazil), who discussed a recombinant BCG vaccine incorporating the LTAK63 adjuvant (rBCG-LTAK63). This candidate enhanced Th1 and Th17 responses and primed the lung environment for rapid immune activation, ^11, 12^ with the circadian rhythm gene Per1 identified as a key factor associated with limiting lung pathology and improving protection in mice by regulating CCR2 expression and restricting GR1+ myeloid cell recruitment. **Ana Carolina Carvalho** (Instituto Butantan, Brazil) presented complementary work on rBCG-LTAK63, demonstrating induction of macrophage autophagy, IL-17 production, and robust T cell activation and effector function in vaccinated mice relative to standard BCG vaccination (unpublished data). **Isabel Lamb-Echegaray** (University of California, Berkeley, USA) shared data on mucosal vaccination with cyclic dinucleotide adjuvants, which demonstrated greater efficacy than BCG and elicited protective IL-17 and IFN-γ responses with T cell homing to the lungs. ^13^ These data highlighted that CDN vaccine efficacy was independent of STAT-6, IRF3, and S365, but dependent on STING and its terminal tail (dCTT). These findings demonstrated that two adjuvants, CDNs and AS01, conferred protection against TB and induced autophagy, while MPLA was not associated with protection. Notably, CDNs induced STING-dependent autophagy which was Beclin1 independent.

### 2.2 Investigate innovative formulations, platforms, and delivery routes

Promising preclinical data were shared on two attenuated *Mtb* mutants as vaccine candidates. **Ramandeep Singh** (THSTI, India) discussed toxin-antitoxin (TA) systems in mutant *Mtb*, highlighting that immunization of animals with TA-deficient *Mtb* strains conferred protection and induction of Th1 and memory T cell responses in guinea pigs and mouse models. ^14, 15^ These data suggest TA systems are important for *Mtb* pathogenicity, and TA-deficient *Mtb* strains may thus serve as future vaccine candidates. **Dhiraj K. Singh** (Texas Biomedical Research Institute, USA) presented on DsigH, an attenuated *Mtb* mutant. Vaccination with DsigH was associated with robust antigen-specific T cell responses in the lung, inducible bronchus-associated lymphoid tissue (iBALT) formation, and more efficient antigen presentation by macrophages to T cells in cynomolgus macaques, corroborating prior studies. ^16– 18^ A favorable safety profile alongside evidence that vaccine-induced protection occurred prior to granuloma formation indicates DsigH as a promising candidate for further development in the context of *Mtb/*HIV co-infection. ^19^

**Elena Stylianou** (University of Oxford, UK) presented data on a heterologous prime-boost subunit vaccine strategy using a combination of five antigens (PPE15, esxI, esxJ, PE8, Ag85A). In mouse models, the ChAdOx-MVA platform improved BCG efficacy in both lung and spleen. ^20^ The same regimen also provided efficacy in non-human primates (NHP), by reducing disseminated disease. **Marcellus Korompis** (University of Oxford, UK) presented PPE15-LMQ, a novel protein adjuvanted formulation, that provided significant protection against *Mtb* in mice, both as a standalone vaccine and as a BCG booster. ^21^ PPE15-LMQ-induced protection was associated with robust CD4 responses in the lungs and spleen and homing of T cells to the lung parenchyma.

**Andy C. Tran** (St. George’s University of London, UK) presented Platform CTB-Fc (PCF), a self-adjuvanting single-polypeptide vaccine platform containing an IgG-Fc fragment, expressed via a recombinant plant system and designed for aerosolized delivery. PCF previously demonstrated immunogenicity and efficacy against Dengue and SARS-CoV2 infections in mice. ^22^ Novel trialing of PCF with ESAT6/CFP10 antigen inserts in *Mtb*-infected mice elicited antibody responses in both serum and lung mucosa, as well as Th1 and Th17 responses. Significant protection was observed in an *ex vivo* modified mycobacterial growth inhibition assay (MGIA) (unpublished data). Further work is planned to investigate other *Mtb* antigens in this platform. **Ross Fulton** (Akagera Medicines, Inc., USA) discussed a lipid nanoparticle-based mRNA vaccine, with promising preclinical efficacy in mice (unpublished data). Success of nanoparticle uptake was attributed to the use of phosphatidyl L-serine in the vaccine formulation, ^23^ highlighting the importance of innovative delivery platforms in improving efficacy.

**Patricia Darrah** (Vaccine Research Center, NIH, USA) shared findings from rhesus macaque studies showing that high-level protection after intravenous (IV) BCG vaccination correlated with *Mtb*-specific T cell responses and NK cells in the airways, ^24^ and that depleting CD4 T cells from IV BCG-immunized animals abrogated protection. ^25^ These data highlight the value of IV BCG as a benchmark to study immune correlates and mechanisms of protection in a pre-clinical model, as well as to inform future vaccine design through mapping responses from IV BCG-vaccinated animals to discover new T cell antigens. **Frank Verreck** (Biomedical Primate Research Centre, Netherlands) presented pilot data on aerosol-inhaled MTBVAC efficacy and a full study report on protection (PoD) by pulmonary mucosal BCG re-vaccination in NHPs, showing complementary robust Th1/Th17 resident memory T cell (T _RM_) responses in the airways compared to conventional intradermal administration. ^26^ Similarly, **Marcela Henao-Temayo** (Colorado State University, USA) demonstrated that SolaV-TB, an intranasal mucosal vaccine derived from *Mtb* (H37Rv strain), inactivated using SolaVAX technology, ^27^ improved bacterial control in the lungs at chronic timepoints post-infection, and elicited *Mtb*-specific antigenic responses when used as a BCG booster (unpublished data).

### 2.3 Adopt optimized animal and human challenge models

A range of efforts are underway to develop robust animal models to elucidate mechanisms of protection to help prioritize and more quickly assess vaccine candidates. **Courtney R. Plumlee** (Seattle Children’s Research Institute, USA) introduced an ultra-low-dose (ULD) *Mtb* murine challenge model (~1 CFU/mouse) that more closely mimics key features of human *Mtb* infection compared to conventional dose murine models (~100 CFU/mouse), helping reveal novel mechanisms of protection. ^28, 29^ Using this model, BCG induced distinct CD4 and CD8 T cell responses, with CD4 T cells associated with controlling bacterial burden and dissemination, and CD8 T cells with preventing *Mtb* infection. Conversely, CD8 T cells were not associated with conferring protection following conventional dose challenge. These data suggest that BCG induces CD4 and CD8 T cells that play complementary roles in protection against TB.

**Helen McShane** (University of Oxford, UK) discussed the value of controlled human infection models (CHIM) in facilitating and de-risking early stage vaccine R&D by providing a meaningful biological signal of efficacy with candidate vaccines. McShane discussed the evaluation of a prior BCG vaccination against intradermal BCG challenge (alone and in combination with the MVA85A vaccine candidate) ^30^ and via aerosol challenge ^31– 33^ in humans as human efficacy data is available for both BCG and MVA85A. The ultimate ‘validation’ of a CHIM would be comparison of candidate vaccine ‘efficacy’ in a CHIM versus a field efficacy study; therefore, it would be useful to evaluate M72/AS01E in these BCG CHIMs. **Anil S. Pooran** (University of Cape Town Lung Institute, South Africa) presented a human antigenic challenge model in the lungs using bronchoscope-delivered BCG and purified protein derivative (PPD). The model was shown to be safe in a study that stratified HIV-uninfected participants into different susceptibility profiles, defined as protective (close TB contacts of TB index cases who did not develop TB) and susceptible (individuals with at least one previous TB episode). ^34^ Additional data revealed distinct lung and blood immune profiles, with upregulated neutrophil, type I IFN, NK cell, and immunoglobulin pathways in susceptible individuals. Further research may help identify candidate CoPs related to protection induced by BCG or other live mycobacterial vaccines.

### 2.4 Host immune mechanisms and host-directed therapies

Understanding and harnessing host immune mechanisms, including as immune correlates, is essential for guiding the design of next-generation TB vaccines.

**G V R Krishna Prasad** (Washington University in St. Louis, USA) described the role of Signaling Lymphocyte Activation Molecule Family Member 1 (SLAMF1), a microbial sensor in macrophages. ^35^ SLAMF1 was required for macrophage-T cell interactions during *Mtb* infection in mice, conferring protection by controlling bacterial burden in macrophages (unpublished data). **Smriti Mehra** (Texas Biomedical Research Institute, USA) further explored host-directed therapies (HDTs), presenting data on 1-methyl-d-tryptophan, an indoleamine dioxygenase (IDO) inhibitor, as an adjunctive therapy in the context of *Mtb* infection ^36^ and combination antiretroviral therapy (cART) in an NHP model. *Mtb*-infected animals receiving both cART and 1-methyl-d-tryptophan had reduced TB pathology and retroviral loads. ^37^ These data also implicate IDO as both an immunoregulatory molecule and potential HDT target.

## 3. Accelerating clinical development

### 3.1 Learning from past trials

Insights from past TB vaccine trials have provided valuable lessons that are shaping strategies for the development of more effective and targeted vaccines for the future. **Thomas Scriba** (University of Cape Town, South Africa) presented findings from the H56:IC31 vaccine trial [ NCT03512249], which aimed to assess efficacy of H56:IC31 to prevent recurrence in people treated for TB. The vaccine was found to be immunogenic. However, an increased rate of TB recurrence was observed in those receiving the vaccine (5.8%) relative to placebo controls (3.4%), although this difference was not statistically significant. ^38^ Additional analysis revealed that, regardless of study arm, the baseline inflammatory state (i.e., before vaccination) of those who experienced recurrence was higher than those who did not experience recurrence (to note, the analysis cannot discriminate between relapse versus reinfection). These data highlight the importance of understanding factors, such as inflammatory responses to previous disease, that could predict relapse. **Alexander Schmidt** (Gates Medical Research Institute (MRI), USA) discussed the outcomes of BCG revaccination trials [unpublished data on NCT04152161]. ^39– 44^ These studies demonstrated that BCG revaccination did not provide significant protection from TB disease or infection. Schmidt underscored that BCG vaccination of infants does prevent TB and should continue, and that results for prevention of disease trials in India using recombinant BCG are anticipated. Schmidt also shared that recruitment is well on track for the M72/AS01E Phase 3 efficacy trial underway in five countries, with a target to enrol 20,000 participants aged 15 to 44 years old [ NCT06062238]. M72/AS01E earlier demonstrated 50% efficacy in preventing active pulmonary TB in a Phase 2b trial. ^45^ **Paulo Cesar Pereira dos Santos** (Universidade Federal de Mato Grosso do Sul, Brazil) presented results from a nested randomized controlled trial within the BRACE trial [ NCT04327206] evaluating the impact of BCG-vaccination against primary or sustained *Mtb* infection, regardless of prior BCG vaccination status. ^39, 46^ The trial failed to demonstrate significant protection of the vaccine and highlighted the need to better understand TB prevention in high-risk populations.

**Ingrid Murillo Jelsbak** (Biofabri, SLU, Spain) provided updates on MTBVAC, a live attenuated TB vaccine, with promising safety and immunogenicity data in neonates, and adolescents and adults in early-stage trials (Phase 1a in adults [ NCT02013245]; Phase 1b [ NCT02729571] and Phase 2a [ NCT03536117] in neonates). A Phase 1b/2a in adults [ NCT02933281] was also carried out in parallel followed by an ongoing Phase 2a [ NCT05947890] in people living with HIV. With two late-stage trials currently underway (Phase 3 in neonates [ NCT04975178]; Phase 2b in adults and adolescents [ NCT06272812]), Jelsbak emphasized the need for capacity-building in low- and middle-income countries (LMICs) to support vaccine production and delivery. **Lisa Deanne Schlehuber** (Gates MRI, USA) discussed the MESA-TB trial [ NCT04556981] results, demonstrating safety and immunogenicity of M72/AS01E in people living with HIV on ART who were virally suppressed (unpublished data). These results highlight the feasibility of including immunocompromised populations, who are often underrepresented, in TB vaccine trials in support of equitable post-licensure access. **Alemnew Dagnew** (Gates MRI, USA) summarized results from the global epidemiological study, TBV02-E01 [NCT05190146], that assessed interferon gamma release assay (IGRA) positivity in high TB burden populations in 14 countries to identify eligible sites for the M72/AS01E Phase 3 trial [ NCT06062238]. IGRA positivity rates varied between countries and sites within a country, and higher IGRA positivity rates correlated with increased age (unpublished data). The findings informed data-driven site selection for the trial, as well as site specific adjustment to recruitment strategies.

### 3.2 Reducing timelines to TB vaccine licensure

**Ann Ginsberg** (Gates Foundation, USA) highlighted key barriers to rapid TB vaccine licensure, including prolonged development timelines due largely to TB epidemiology, the lack of CoP and the current use of clinical TB disease as the endpoint for licensure. **Claudia Crowell** (BioNTech, Germany) discussed approaches to optimize trial design to improve efficiency and reduce development timelines, including; (i) refined endpoint selection through critical evaluation of the definition of clinical TB (cTB), (ii) implementing adaptive trial designs to adjust trials based on interim findings, (iii) flexible trial designs that overlap trial phases to save time, and (iv) engaging with regulatory authorities early to enable smooth transitions between trial phases. **Frank Cobelens** (Amsterdam Institute for Global Health and Development, Netherlands) showed, based on mathematical modeling, the effect of only including IGRA-positive participants versus no pre-enrolment IGRA screening on the required sample size for late stage efficacy trials. Findings suggested that trials including only IGRA-positive participants can have considerably smaller sample sizes in settings where the TB incidence is relatively low (e.g., East Africa) but this efficiency gain is lost in settings where the TB incidence is relatively high (e.g., South Africa).

**Elana van Brakel** (IAVI, South Africa) highlighted promising candidates in late-stage PoD trials that are contributing samples to biorepositories for identifying CoP as one of the greatest strengths of the clinical development pipeline, while the inverted nature of the current pipeline remains a striking weakness. Despite new vaccine platforms, such as mRNA constructs, in early-stage trials, there are relatively few novel candidates in early clinical development. Accelerated development requires more candidates entering clinical development but also eliminating unsuccessful candidates at earlier stages before expensive, scaled-up efficacy trials. Van Brakel further reflected on whether trial endpoints can be differentially evaluated to assess if a vaccine being tested for one end point, such as Prevention of Infection (PoI), could be considered for another, such as PoD or Prevention of Recurrence (PoR). ^47– 49^ **Gavin Churchyard** (Aurum Institute, South Africa) explored the advantages and pitfalls of a composite endpoint that includes subclinical TB (scTB) and cTB in PoD trials, ^49^ illustrating a range of study designs with varying sample sizes and diagnostic timepoints. Churchyard noted however that if a vaccine has differential efficacy, a vaccine efficacious in preventing cTB may inadvertently be rejected if found to be ineffective against scTB. More evidence is needed to support including scTB as part of a composite endpoint.

**Puck Pelzer** (IAVI, Netherlands) presented an individual patient data meta-analysis, evaluating the effectiveness of primary BCG against the risk of Mtb infection and TB disease ^50^ to explore how PoI translates to PoD. QuantiFERON-TB (QFT) conversion only demonstrated protection against disease in groups with recent household exposure. The predictive effect for PoD of Tuberculin Skin Test (TST) conversion lacks value for assessing BCG prevention of infection as well as disease. Future research among subpopulations is required to determine whether QFT testing could serve as a proxy for disease for late-stage TB vaccine trials. **Simon Mendelsohn** (University of Cape Town, South Africa) reported the performance of blood mRNA signatures for predicting TB relapse among 111 adults with drug-susceptible TB in South Africa and Tanzania enrolled in a PoR trial of the H56:IC31 vaccine candidate in South Africa and Tanzania ( NCT03512249). The analysis demonstrated that individuals who relapsed had higher mRNA signature scores, synonymous with persistent systemic inflammation, in the final month of treatment, suggesting unsuccessful pathogen clearance and/or ongoing immune activation (unpublished data) – mRNA signatures could thus be used to identify and monitor individuals at greatest risk of relapse. Signature scores and predictive performance did not differ between H56:IC31 or placebo recipients.

### 3.3 Correlates of protection: the way forward

Identifying robust correlates of vaccine-induced protection has the potential to inform vaccine design and reduce the length and cost of future clinical trials through early identification of immunological markers. However, it remains challenging to define CoP across diverse TB populations and vaccine types.

**Elisa Nemes** (University of Cape Town, South Africa) shared perspectives and ongoing analyses on CoP ^51^ and correlates of risk (CoR) from recently completed clinical trials of BCG revaccination for PoI, ^40^ M72/AS01E for PoD ^52^ and H56/IC31 for PoR. ^38^ Nemes highlighted the importance of sample type and endpoint selection in human trials to identify meaningful CoP across different vaccine candidates and clinical end-points. Most clinical research to date has focused on human peripheral blood for identifying CoP. Although useful, these data do not necessarily represent tissue-level immune responses at sites of *Mtb* infection or niches of persistence, suggesting a need to study compartments such as the lungs and lymph nodes for tissue-specific immune responses to complement peripheral blood data. For example, **Shuailin Li** (University of Oxford, UK) demonstrated that the enrichment of lungs with antigen specific CD4 T cells in vaccinated mice, elicited through protein-adjuvanted subunit vaccines, were crucial for stronger TB protection. ^21^ **Iman Satti** (University of Oxford, UK) noted that protection against TB disease and *Mtb* infection is likely driven by distinct immune mechanisms. ^53, 54^ Satti emphasized the importance of CHIM studies, which facilitate the identification of CoP and allow for the characterization of immune responses following timed mycobacterial infections. Additionally, Satti highlighted the value of *ex vivo* growth inhibition assays using samples from human and NHPs to bridge between species, facilitating the assessment of protective immune mechanisms and vaccine efficacy. ^55^

**Carly Young** (University of Cape Town, South Africa) presented preliminary data of *Mtb* nucleic acid detection in the lymph nodes of 67% of otherwise healthy individuals without TB pathology at autopsy (total n=69) (unpublished data), highlighting the systemic nature of *Mtb* infection and potential role of lymphatic niches in *Mtb* persistence. Further research is needed to better understand niches of persistence, the associated host immune responses and potential immune correlates, and how they may influence the outcome of infection. Reflecting on the valuable immunological insights offered by organoid models, **Elly van Riet** (TBVI, Netherlands) noted that these models could help fill gaps posed by the lack of both correlates of protection and models predictive for human efficacy. However, current models may not fully capture the complexity of TB immunity in humans, underscoring the need to further optimize these models and select the preclinical model best suited for the questions under investigation.

Immune responses elicited by TB vaccines vary based on platform (i.e., live attenuated, live inactivated, protein subunit, viral vector, or RNA). **Elizabeth Church** (Fred Hutchinson Cancer Center, USA) emphasized that due to the complexity of immune responses to TB, a single CoP is not likely to be universally applicable across all vaccine types. Further research is needed to better understand these variations and identify reliable CoP for different vaccine platforms. **Gavin Churchyard** (Aurum Institute, South Africa) highlighted that approved CoP would enable immune-bridging studies to expand licensure across populations and potential biomarkers could help stratify individuals for targeted interventions, enhancing trial design and vaccine implementation strategies. Churchyard further suggested that multiple assays will likely be needed to define CoP and recommended that TB vaccine trials prioritize storage of specimens to support further development of CoP.

## 4. Ensuring public health impact and preparing for implementation

Successful implementation of new TB vaccines requires preparation, collaboration, and sustained financing among multiple stakeholders in advance of licensure to develop policy pathways, manufacturing plans, and procurement strategies for rapidly scaled-up introduction. Aspects of national and global level implementation planning, including regulatory affairs, financing, and partnership models were discussed throughout the Forum.

### 4.1 Strategic planning for TB vaccine implementation

Ensuring adequate supply is crucial to mitigate challenges such as shortages, introduction delays, prohibitive costs, and manufacturing sustainability. **Birgitte Giersing** (WHO, Switzerland) discussed the need to carefully consider the interplay between country and global level activities, noting that self-procuring countries are potential early adopters and thus likely the first to generate real world data on new TB vaccines. ^56^ Reflecting on this, **Shelly Malhotra** (IAVI, USA) highlighted the need to understand country-specific implementation scenarios that reflect a country’s context and delivery infrastructure, including procurement pathways (i.e., domestic versus Gavi-funded), target populations (i.e., adults, adolescents, infants), and priority use cases, noting that vaccine characteristics will be a key driver of uptake. ^57^ This information can help inform understanding of demand and support supply planning to ensure manufacturing capacity is scaled accordingly, a process which can take several years. **Marta Tufet Bayona** (Gavi, Switzerland) provided an overview of Gavi’s Vaccine Investment Strategy (VIS), which assesses the impact, cost, and feasibility of new or underused vaccines for Gavi-eligible countries every five years. ^58^ Through evidence-based analysis and extensive consultations, the VIS sets priorities for Gavi’s vaccine support programs. In June 2024, the Gavi Board approved a new VIS. This in-principle approval allows Gavi to begin market signaling and preparedness activities in advance, and includes a learning agenda and potential expansion of Gavi’s portfolio to include a new TB vaccine for adults and adolescents once recommended for use and prequalified by WHO. Tufet Bayona highlighted the need for partners and stakeholders to work closely together to ensure that any TB vaccine is accessible to those who need it, as part of a comprehensive, integrated approach to prevention, diagnosis, and care.

Preliminary modelling presented by **Rebecca A. Clark** (LSHTM, UK) compared supply constraint scenarios (20, 50, 100, or 150 million doses per year) of a 50% efficacious vaccine in preventing disease delivered routinely to individuals aged 15 years with 80% coverage from 2030–2050 in 105 countries. Under supply constraints, prioritizing countries with highest TB incidence first will avert more episodes of TB (unpublished data). This study underscores the need for accurate demand forecasts to inform manufacturing capacity and mitigate supply issues. **Fortune Benjamin Effiong** (Medical Laboratory Science, Nigeria) summarized that novel TB vaccines targeting adolescents and adults are likely to be cost-effective in LMICs compared to BCG based on insights from five modelling studies, ^59– 63^ with cost-effectiveness varying depending on vaccination strategy and increasing with greater vaccine efficacy, duration of protection, and coverage level.

Discussions on country preparedness efforts provided insights on how high burden countries are already laying the groundwork for the implementation of new TB vaccines. **Norbert Ndjeka** (National Department of Health, South Africa) described the creation of a TB vaccine working group in South Africa to collect evidence and provide guidance for TB vaccine introduction. Ndjeka highlighted opportunities to harmonize introduction across Africa through coordinating with and aligning data needs of regional regulators via the African Vaccine Regulatory Forum and expanding capacity of regional manufacturers. **Thayssa Victer** (Ministry of Health, Brazil) described the close alignment between multiple national agencies necessary for effective vaccine procurement in Brazil, including the Brazilian Health Regulatory Agency (ANVISA), National Health Fund (FNS), and the National Commission for the Incorporation of Technologies into the Unified Health System (CONITEC). Victer further highlighted Brazil’s role as a vaccine manufacturing hub in the PAHO region but added that while local manufacturing in Brazil is important, introducing a new vaccine as quickly as possible is a higher priority, even if not initially produced in-country. **Jessy Joseph** (IAVI, India) described a comprehensive, integrated TB vaccine modelling framework from India which has brought together key partners including ICMR-National Institute for Research in Tuberculosis (NIRT), Ministry of Health and Family Welfare, Department of Biotechnology, WHO, and IAVI. Informed through iterative multi-stakeholder engagement, the “living” model can generate nationally relevant evidence to optimize planning for vaccine rollout in the country. Demonstrative scenarios predicted burden reduction through PoI, PoD, and PoR vaccines across different age groups and risk groups and synergistic effects of vaccination with other preventive measures, such as nutritional interventions (unpublished data).

### 4.2 Perspectives on scale up and delivery

Robust governmental support, regulatory processes, and programmatic planning are critical to mitigate barriers in TB vaccine commercialization and delivery. **Richard G. White** (LSHTM, UK) emphasized the importance of applying lessons from COVID-19 and malaria vaccines for scaling up TB vaccine delivery, advocating for robust and coordinated international efforts to achieve sufficient scale. **Rosane Cuber Guimarães** (Bio-Manguinhos-Fiocruz, Brazil) considered manufacturing perspectives, highlighting challenges such as the lack of reliable biomarkers, limited infrastructure, and hesitant industry engagement due to uncertain demand. Cuber Guimarães stressed the importance of strong governmental commitments and leveraging North-South and South-South partnerships to mitigate these challenges and enhance equitable access. **Eder Gatti Fernandes** (National Immunization Program, Brazil) called for vaccines with long shelf lives to minimize waste, underscoring the importance of innovative cold-chain logistics to overcome distribution challenges. Gatti Fernandes noted that this will likewise require substantial investments and global coordination.

### 4.3 Reaching key and vulnerable populations

Generating demand and acceptance is critical to ensure uptake of new TB vaccines. **Andrew D. Kerkhoff** (University of California San Francisco, USA) reported high levels of intention to receive a TB vaccine among community members (76.9% (95% CI: 71.9-82.0)) and healthcare workers (HCWs) (82.6% [95% CI: 71.5–93.6]) in Lusaka, Zambia (unpublished data). Intention to vaccinate was strongly associated with perceived TB risk, ranging from 49.5% (22.6–76.5) to 82.4% (77.6–87.2) among those “not at all concerned” to “very concerned” about TB, respectively. The Ministry of Health (87.6%), HCWs (68.2%), and the government (53.8%) were found to be the most trusted sources for vaccines. **Kinz ul Eman** (Dopasi Foundation, Pakistan) showcased how community-driven implementation of TB preventive therapy (TPT) in nine districts in Pakistan successfully initiated 5,423 people (7,747 eligible) on TPT over 12 months, compared to 362 in the preceding 12 months. The initiative combined advocacy and capacity building with a digital solution to effectively overcome challenges in TPT, including around acceptance, with relevant lessons for future TB vaccine deployment (unpublished data).

Reflecting on how the inclusion of key populations in TB vaccine research is ethically feasible, **Rupali Limaye** (Johns Hopkins Bloomberg School of Public Health, USA) called for transparent frameworks to maximize health impact and equitable access, particularly in high-burden areas, while maintaining rigorous ethical standards. Limaye acknowledged that safety data for certain populations might however depend on post-licensure trials. **Edna Tembo** (Coalition of Women Living with HIV and AIDS (COWLHA), Malawi) discussed the community consensus on the inclusion of pregnant and breastfeeding women in TB vaccine trials, highlighting the importance of giving key populations the necessary information and capacity to choose whether to participate. ^64^ **Crhistinne Cavalheiro Maymone Gonçalves** (Brazil) and **Monica Magalhaes** (Rutgers, USA) emphasized the need to uphold ethical standards without compromising involvement of vulnerable populations, discussing how persons deprived of liberty can be ethically included with appropriate safeguards to maintain confidentiality and prevent coercion. **Martha Nyakambi** (PHDA, Kenya) called for the integration of TB vaccines into mainstream services and reduction of stigma through community-driven awareness programs. **Peter Ngo’la Owiti** (Stop TB Partnership, Kenya) stressed that TB vaccines must be easy to administer at the nearest point of contact immediately upon initiation of rollout, while highlighting the role of community ambassadors in mitigating misinformation.

## 5. Enabling conditions for TB vaccine development

Ensuring progress in TB vaccine R&D requires a range of enabling conditions, including fulfilling political commitments, mobilizing increased and sustained finances and resources, open science, and community engagement.

### 5.1 Political commitment and funding

Reflecting on the targets endorsed in the Political Declaration of the 2023 UN HLM on TB, **Shaun Palmer** (IAVI, Netherlands) called for advocates to present proposals to public sector and other funding bodies on approaches they can take to finance TB vaccine R&D in a coordinated and sustained way, highlighting the opportunity for MICs to lead innovative financing initiatives. ^65^ **Deborah King** (Wellcome, UK) noted that philanthropies can lead by example—taking on higher risk investments for global health and working in partnership to share the risk. King further reflected on how investments from philanthropies can incentivize sustainable investments from other sources and sectors, as previously seen with initiatives such as CEPI and the AMR Action Fund. ^66, 67^ **Mike Frick** (Treatment Action Group, USA) shared a call to re-focus on national advocacy work and engage directly with governments; urging civil society to maintain a united front in this effort and leverage diverse skills and perspectives. **Leigh Raithby** (Results Canada, Canada) underscored the importance of advocacy in high-income countries to mitigate political divisions, underfunding, and competition with other health priorities. Raithby added that education of decision makers on the need and potential solutions will be crucial to hold leaders accountable to their commitments. **Erin V. McConnell** (Treatment Action Group, USA) offered insights on how, following a rise in sovereign debt burdens from 40% to 60% of GDP in 44 LMICs between 2011 and 2020, ^68^ equitable financing approaches such as debt relief, concessional financing, Special Drawing Rights, and debt legislative reform and restructuring can mitigate debt distress and create domestic fiscal space to prepare for TB vaccine development and deployment, alongside other priority health areas. ^69^

### 5.2 Open science and collaboration

**Soumya Swaminathan** (M S Swaminathan Research Foundation, India) reflected on the development of target product profiles, timely data sharing, voluntary tech transfer, and public-private partnerships during the COVID-19 pandemic that enabled successful vaccine implementation. Looking ahead, streamlining pathways to accelerate development will be key, including harmonized trial protocols, standardized assays, and expedited regulatory approvals. Swaminathan called for global mechanisms for collaborative decision-making and open science to support comprehensive data repositories, large-scale clinical testing, market shaping, and evidence-based decisions rooted in community based partnerships, with equity at the heart of vaccine development. **Gerald Voss** (TBVI, Netherlands) called for more innovative approaches in TB vaccine development, such as head-to-head comparisons of vaccine candidates, alongside platform synergies to encourage industry investment and evidence-based business cases to support market access. Concerning open science, Voss noted that negative data should be published to ensure access to all relevant information. **Carly Young** (SATVI, University of Cape Town, South Africa) highlighted the need to attract and retain early career researchers in the TB vaccine field through efforts such as diverse professional development opportunities, the ability to maintain ownership of their work, fairer compensation, and more sustainable funding opportunities to avoid attrition from the field. **Erica Chimara** (Rede-TB, Brazil) spoke about the need for greater unity across the TB vaccine field. Chimara reflected on REDE-TB’s experience, including an ongoing effort to form a Latin American regional network for TB research involving academia, government, health services, and civil society. **Jackie Cuen** (We are TB/Somos TB, USA) stressed that good science requires robust inclusion, collaboration, and communication, including between researchers and advocates, which can help better translate science into clinical impact. **David Lewinsohn** (Stop TB Partnership Working Group on New TB Vaccines, USA) further emphasized the value of researchers engaging in advocacy and collaborating closely with community members, before sharing a general call for the global TB community to harness its collective outrage to drive policy change and increase funding.

### 5.3 Effective community engagement

**Keyuri Bhanushali** (Survivors Against TB, India) called for interactive community engagement, including local and religious leaders, across the research to implementation continuum to focus research strategies, foster trust, and respond to real-world community needs. **Doreen Ibrahim Pamba** (National Institute for Medical Research, Tanzania) noted that community sensitization on TB vaccine research is crucial throughout this process. **Ezio Távora** (REDE-TB, Brazil) added that research literacy efforts are a core aspect of this approach while noting that trials should be conducted in globally representative, high burden regions, including Latin America. **Patrick Agbassi** (Global TB CAB, Côte d’Ivoire) and **Carla Almeida** (Brazilian TB CAB, Brazil) stressed that studies must budget for sustained community engagement from the outset, and **Phorng Chanthorn** (Khmer HIV/AIDS NGO Alliance (KHANA), Cambodia) called for dedicated investments in terms of financial assistance and operational structures to strengthen grassroots movements in high-burden regions and build upon the leadership of TB survivors and facilitate implementation.

## 6. Conclusion

The 7th Global Forum on TB Vaccines showcased the promising research taking place across the breadth of the field and underscored the need for greater innovation and investments to advance clinical development and ensure rapid, affordable, and equitable rollout. While late-stage clinical trials of the M72/AS01E and MTBVAC candidates are underway, with results anticipated as early as 2028, urgent priorities remain, including the diversification of the preclinical and clinical pipelines, the identification of immune CoP, and the optimization of R&D practices, all underpinned by open and transparent science. Novel financing and partnership models based on global, multilateral partnerships with enhanced leadership from HBCs are likewise needed. Related capacity strengthening in HBCs, including in trial site capacity, regulatory affairs, and localized manufacturing, will help achieve equitable access. Inclusive collaboration with affected community members and civil society that incorporates the needs and perspectives of key and vulnerable populations should be prioritized at all stages of development and implementation to inform responsive R&D and foster trust and ownership of TB vaccines once licensed. Coordinated advocacy at the grassroots and global level will be crucial to hold governments accountable to their commitments on TB vaccines and mobilize the necessary resources to make new TB vaccines a reality this decade.

## Author contributions

All authors have contributed to, reviewed, and approved the final, submitted version of the manuscript. All authors attest they meet the ICMJE criteria for authorship.

## Consent for publication

All individuals mentioned in the manuscript and the acknowledgement section consented to the inclusion of their name and affiliation in the report.

## Disclaimer

The views expressed in this article are those of the authors. Publication in Gates Open Research and the VeriXiv preprint server does not imply endorsement by the Gates Foundation or the United States Government.

## Data Availability

No data was used for the research described in the article.

## References

[1] Ghebreyesus T Trindade LN : The TB Vaccine Accelerator Council: Harnessing the power of vaccines to end the tuberculosis epidemic. *Lancet Infect. Dis.* 2023;23:1222–1223. 10.1016/S1473-3099(23)00589-3 37742697

[2] United Nations: *Political Declaration on the High-Level Meeting on the Fight Against Tuberculosis.* United Nations;2023.

[3] Community Engagement Committee of the 7th Global Forum on TB Vaccines: Community Declaration of the 7th Global Forum on TB Vaccines. 2024. (accessed February 17, 2025). Reference Source

[4] Ogongo P Wassie L Tran A : Rare Variable M. tuberculosis Antigens induce predominant Th17 responses in human infection. *BioRxiv.* 2024. 10.1101/2024.03.05.583634 38496518 PMC10942433

[5] Ogongo P Tezera LB Ardain A : Tissue-resident-like CD4+ T cells secreting IL-17 control Mycobacterium tuberculosis in the human lung. *J. Clin. Invest.* 2021;131:131. 10.1172/JCI142014 33848273 PMC8121523

[6] Scriba TJ Penn-Nicholson A Shankar S : Sequential inflammatory processes define human progression from M. tuberculosis infection to tuberculosis disease. *PLoS Pathog.* 2017;13:e1006687. 10.1371/JOURNAL.PPAT.1006687 29145483 PMC5689825

[7] Nathan A Beynor JI Baglaenko Y : Multimodally profiling memory T cells from a tuberculosis cohort identifies cell state associations with demographics, environment and disease. *Nat. Immunol.* 2021;22:781–793. 10.1038/S41590-021-00933-1 34031617 PMC8162307

[8] Mayer RL Impens F : Immunopeptidomics for next-generation bacterial vaccine development. *Trends Microbiol.* 2021;29:1034–1045. 10.1016/J.TIM.2021.04.010 34030969

[9] Leddy O White FM Bryson BD : Immunopeptidomics reveals determinants of Mycobacterium tuberculosis antigen presentation on MHC class I. *elife.* 2023;12:12. 10.7554/ELIFE.84070 37073954 PMC10159623

[10] Panda S Morgan J Cheng C : Identification of differentially recognized T cell epitopes in the spectrum of Mtb infection. *BioRxiv.* 2023. 10.1101/2023.04.12.536550 38278794 PMC10817963

[11] Nascimento IP Rodriguez D Santos CC : Recombinant BCG Expressing LTAK63 Adjuvant induces Superior Protection against Mycobacterium tuberculosis. *Sci. Rep.* 2017;7:2109. 10.1038/S41598-017-02003-9 28522873 PMC5437048

[12] Marques-Neto LM Trentini MM Kanno AI : Recombinant BCG expressing the LTAK63 adjuvant increased memory T cells and induced long-lasting protection against Mycobacterium tuberculosis challenge in mice. *Front. Immunol.* 2023;14:14. 10.3389/FIMMU.2023.1205449 37520577 PMC10374402

[13] Jong RM Van Dis E Berry SB : Mucosal Vaccination with Cyclic Dinucleotide Adjuvants Induces Effective T Cell Homing and IL-17-Dependent Protection against Mycobacterium tuberculosis Infection. *J. Immunol.* 2022;208:407–419. 10.4049/JIMMUNOL.2100029 34965963 PMC8755605

[14] Gosain TP Singh M Singh C : Disruption of MenT2 toxin impairs the growth of Mycobacterium tuberculosis in guinea pigs. *Microbiology (Reading).* 2022;168:168. 10.1099/MIC.0.001246 36342835

[15] Gosain TP Chugh S Rizvi ZA : Mycobacterium tuberculosis strain with deletions in menT3 and menT4 is attenuated and confers protection in mice and guinea pigs. *Nat. Commun.* 2024;15:5467. 10.1038/S41467-024-49246-5 38937463 PMC11211403

[16] Mehra S Golden NA Stuckey K : The Mycobacterium tuberculosis stress response factor SigH is required for bacterial burden as well as immunopathology in primate lungs. *J. Infect. Dis.* 2012;205:1203–1213. 10.1093/INFDIS/JIS102 22402035 PMC3308902

[17] Dutta NK Mehra S Martinez AN : The stress-response factor SigH modulates the interaction between Mycobacterium tuberculosis and host phagocytes. *PLoS One.* 2012;7:7. 10.1371/JOURNAL.PONE.0028958 PMC3250399 22235255

[18] Kaushal D Foreman TW Gautam US : Mucosal vaccination with attenuated Mycobacterium tuberculosis induces strong central memory responses and protects against tuberculosis. *Nat. Commun.* 2015;6:8533. 10.1038/NCOMMS9533 26460802 PMC4608260

[19] Singh DK Ahmed M Akter S : Prevention of tuberculosis in cynomolgus macaques by an attenuated Mycobacterium tuberculosis vaccine candidate. *Nat. Commun.* 2025;16:1920–1957. 10.1038/s41467-025-57090-4 40000643 PMC11861635

[20] Stylianou E Pinpathomrat N Sampson O : A five-antigen Esx-5a fusion delivered as a prime-boost regimen protects against M.tb challenge. *Front. Immunol.* 2023;14:14. 10.3389/FIMMU.2023.1263457 37869008 PMC10585038

[21] Korompis M De Voss CJ Li S : Strong immune responses and robust protection following a novel protein in adjuvant tuberculosis vaccine candidate. *Sci. Rep.* 2025;15:1886. 10.1038/S41598-024-84667-8 39805855 PMC11729893

[22] Kim MY Vergara E Tran A : Marked enhancement of the immunogenicity of plant-expressed IgG-Fc fusion proteins by inclusion of cholera toxin non-toxic B subunit within the single polypeptide. *Plant Biotechnol. J.* 2024;22:1402–1416. 10.1111/PBI.14275 38163285 PMC11022806

[23] Tenchov R Bird R Curtze AE : Lipid Nanoparticles─From Liposomes to mRNA Vaccine Delivery, a Landscape of Research Diversity and Advancement. *ACS Nano.* 2021;15:16982–17015. 10.1021/ACSNANO.1C04996 34181394

[24] Darrah PA Zeppa JJ Wang C : Airway T cells are a correlate of i.v. Bacille Calmette-Guerin-mediated protection against tuberculosis in rhesus macaques. *Cell Host Microbe.* 2023;31:962–977.e8. 10.1016/J.CHOM.2023.05.006 37267955 PMC10355173

[25] Simonson AW Zeppa JJ Bucsan AN : Intravenous BCG-mediated protection against tuberculosis requires CD4+ T cells and CD8α+ lymphocytes. *J. Exp. Med.* 2025;222:222. 10.1084/JEM.20241571 39912921 PMC11801270

[26] Dijkman K Sombroek CC Vervenne RAW : Prevention of tuberculosis infection and disease by local BCG in repeatedly exposed rhesus macaques. *Nat. Med.* 2019;25:255–262. 10.1038/S41591-018-0319-9 30664782

[27] Ragan IK Hartson LM Dutt TS : A Whole Virion Vaccine for COVID-19 Produced via a Novel Inactivation Method and Preliminary Demonstration of Efficacy in an Animal Challenge Model. *Vaccines (Basel).* 2021;9:9. 10.3390/VACCINES9040340 33916180 PMC8066708

[28] Plumlee CR Barrett HW Shao DE : Assessing vaccine-mediated protection in an ultra-low dose Mycobacterium tuberculosis murine model. *PLoS Pathog.* 2023;19:e1011825. 10.1371/JOURNAL.PPAT.1011825 38011264 PMC10703413

[29] Plumlee CR Duffy FJ Gern BH : Ultra-low Dose Aerosol Infection of Mice with Mycobacterium tuberculosis More Closely Models Human Tuberculosis. *Cell Host Microbe.* 2021;29:68–82.e5. 10.1016/J.CHOM.2020.10.003 33142108 PMC7854984

[30] Harris SA Meyer J Satti I : Evaluation of a human BCG challenge model to assess antimycobacterial immunity induced by BCG and a candidate tuberculosis vaccine, MVA85A, alone and in combination. *J. Infect. Dis.* 2014;209:1259–1268. 10.1093/INFDIS/JIT647 24273174 PMC3969545

[31] Satti I Marshall JL Harris SA : Safety of a controlled human infection model of tuberculosis with aerosolised, live-attenuated Mycobacterium bovis BCG versus intradermal BCG in BCG-naive adults in the UK: A dose-escalation, randomised, controlled, phase 1 trial. *Lancet Infect. Dis.* 2024;24:909–921. 10.1016/S1473-3099(24)00143-9 38621405 PMC7618001

[32] Fredsgaard-Jones T Harris SA Morrison H : A dose escalation study to evaluate the safety of an aerosol BCG infection in previously BCG-vaccinated healthy human UK adults. *Front. Immunol.* 2024;15:15. 10.3389/FIMMU.2024.1427371 39611145 PMC11602284

[33] Balasingam S Dheda K Fortune S : Review of Current Tuberculosis Human Infection Studies for Use in Accelerating Tuberculosis Vaccine Development: A Meeting Report. *J. Infect. Dis.* 2024;230:e457–e464. 10.1093/INFDIS/JIAE238 38709726 PMC11326834

[34] Davids M Pooran A Hermann C : A Human Lung Challenge Model to Evaluate the Safety and Immunogenicity of PPD and Live Bacillus Calmette-Guérin. *Am. J. Respir. Crit. Care Med.* 2020;201:1277–1291. 10.1164/RCCM.201908-1580OC 31860339

[35] Barbero AM Trotta A Genoula M : SLAMF1 signaling induces Mycobacterium tuberculosis uptake leading to endolysosomal maturation in human macrophages. *J. Leukoc. Biol.* 2021;109:257–273. 10.1002/JLB.4MA0820-655RR 32991756

[36] Singh B Moodley C Singh DK : Inhibition of indoleamine dioxygenase leads to better control of tuberculosis adjunctive to chemotherapy. *JCI Insight.* 2023;8. 10.1172/JCI.INSIGHT.163101 36692017 PMC9977315

[37] Singh B Sharan R Ravichandran G : Indoleamine-2,3-dioxygenase inhibition improves immunity and is safe for concurrent use with cART during Mtb/SIV coinfection. *JCI Insight.* 2024;9. 10.1172/JCI.INSIGHT.179317 39114981 PMC11383603

[38] Borges ÁH Russell M Tait D : Immunogenicity, safety, and efficacy of the vaccine H56:IC31 in reducing the rate of tuberculosis disease recurrence in HIV-negative adults successfully treated for drug-susceptible pulmonary tuberculosis: A double-blind, randomised, placebo-controlled, phase 2b trial. *Lancet Infect. Dis.* 2025. 10.1016/S1473-3099(24)00814-4 40056922

[39] Santos PCP dos Messina NL Oliveira RD de : Effect of BCG vaccination against Mycobacterium tuberculosis infection in adult Brazilian health-care workers: a nested clinical trial. *Lancet Infect. Dis.* 2024;24:594–601. 10.1016/S1473-3099(23)00818-6 38423021 PMC11111441

[40] Nemes E Geldenhuys H Rozot V : Prevention of M. tuberculosis Infection with H4:IC31 Vaccine or BCG Revaccination. *N. Engl. J. Med.* 2018;379:138–149. 10.1056/NEJMOA1714021/SUPPL_FILE/NEJMOA1714021_DISCLOSURES.PDF 29996082 PMC5937161

[41] Glynn JR Fielding K Mzembe T : BCG re-vaccination in Malawi: 30-year follow-up of a large, randomised, double-blind, placebo-controlled trial. *Lancet Glob. Health.* 2021;9:e1451–e1459. 10.1016/S2214-109X(21)00309-0 34534489 PMC8459381

[42] Barreto ML Rodrigues LC Cunha SS : Design of the Brazilian BCG-REVAC trial against tuberculosis: A large, simple randomized community trial to evaluate the impact on tuberculosis of BCG revaccination at school age. *Control. Clin. Trials.* 2002;23:540–553. 10.1016/S0197-2456(02)00216-7 12392870

[43] Barreto ML Pereira SM Pilger D : Evidence of an effect of BCG revaccination on incidence of tuberculosis in school-aged children in Brazil: Second report of the BCG-REVAC cluster-randomised trial. *Vaccine.* 2011;29:4875–4877. 10.1016/J.VACCINE.2011.05.023 21616115

[44] Karonga Prevention Trial Group: Randomised controlled trial of single BCG, repeated BCG, or combined BCG and killed Mycobacterium leprae vaccine for prevention of leprosy and tuberculosis in Malawi. *Lancet.* 1996;348:17–24. 10.1016/S0140-6736(96)02166-6 8691924

[45] Tait DR Hatherill M Van Der Meeren O : Final Analysis of a Trial of M72/AS01 E Vaccine to Prevent Tuberculosis. *N. Engl. J. Med.* 2019;381:2429–2439. 10.1056/NEJMOA1909953/SUPPL_FILE/NEJMOA1909953_DATA-SHARING.PDF 31661198

[46] Pittet LF Messina NL Orsini F : Randomized Trial of BCG Vaccine to Protect against Covid-19 in Health Care Workers. *N. Engl. J. Med.* 2023;388:1582–1596. 10.1056/NEJMOA2212616/SUPPL_FILE/NEJMOA2212616_DATA-SHARING.PDF 37099341 PMC10497190

[47] Cobelens F Suri RK Helinski M : Accelerating research and development of new vaccines against tuberculosis: A global roadmap. *Lancet Infect. Dis.* 2022;22:e108–e120. 10.1016/S1473-3099(21)00810-0 35240041 PMC8884775

[48] Garcia-Basteiro AL White RG Tait D : End-point definition and trial design to advance tuberculosis vaccine development. *Eur. Respir. Rev.* 2022;31:220044. 10.1183/16000617.0044-2022 35675923 PMC9488660

[49] Churchyard GJ Houben RMGJ Fielding K : Implications of subclinical tuberculosis for vaccine trial design and global effect. *Lancet Microbe.* 2024;5:100895. 10.1016/S2666-5247(24)00127-7 38964359 PMC11464400

[50] Pelzer PT Stuck L Martinez L : Effectiveness of the primary Bacillus Calmette-Guérin vaccine against the risk of Mycobacterium tuberculosis infection and tuberculosis disease: A meta-analysis of individual participant data. *Lancet Microbe.* 2025;6:100961. 10.1016/J.LANMIC.2024.100961 39709975 PMC12778190

[51] Nemes E Fiore-Gartland A Boggiano C : The quest for vaccine-induced immune correlates of protection against tuberculosis. *Vaccine Insights.* 2022;1:165–181. 10.18609/VAC/2022.027 37091190 PMC10117634

[52] Van Der Meeren O Hatherill M Nduba V : Phase 2b Controlled Trial of M72/AS01E Vaccine to Prevent Tuberculosis. *N. Engl. J. Med.* 2018;379:1621–1634. 10.1056/NEJMOA1803484 30280651 PMC6151253

[53] Fletcher HA Snowden MA Landry B : T-cell activation is an immune correlate of risk in BCG vaccinated infants. *Nat. Commun.* 2016;7:7. 10.1038/NCOMMS11290 27068708 PMC4832066

[54] Satti I Wittenberg RE Li S : Inflammation and immune activation are associated with risk of Mycobacterium tuberculosis infection in BCG-vaccinated infants. *Nat. Commun.* 2022;13:6594. 10.1038/S41467-022-34061-7 36329009 PMC9632577

[55] Tanner R Hoogkamer E Bitencourt J : The in vitro direct mycobacterial growth inhibition assay (MGIA) for the early evaluation of TB vaccine candidates and assessment of protective immunity: A protocol for non-human primate cells. *F1000Res.* 2021;10:257. 10.12688/F1000RESEARCH.51640.2 33976866 PMC8097740

[56] World Health Organization: WHO global framework to prepare for country introduction of new TB vaccines for adults and adolescents. 2024.

[57] Clark RA Young C Palmer S : Bridging the gap: Evaluating high TB burden country data needs to support the potential introduction of TB vaccines for adolescents and adults: A workshop report. *Frontiers in Tuberculosis.* 2024;2. 10.3389/ftubr.2024.1384036 PMC13494980 42638638

[58] Gavi: Vaccine Investment Strategy 2024. 2024. (accessed March 10, 2025). Reference Source

[59] Harris RC Quaife M Weerasuriya C : Cost-effectiveness of routine adolescent vaccination with an M72/AS01E-like tuberculosis vaccine in South Africa and India. *Nat. Commun.* 2022;13:602. 10.1038/s41467-022-28234-7 35105879 PMC8807591

[60] Portnoy A Clark RA Quaife M : The cost and cost-effectiveness of novel tuberculosis vaccines in low- and middle-income countries: A modeling study. *PLoS Med.* 2023;20:e1004155. 10.1371/journal.pmed.1004155 36693081 PMC9873163

[61] Jayawardana S Weerasuriya CK Pelzer PT : Feasibility of novel adult tuberculosis vaccination in South Africa: A cost-effectiveness and budget impact analysis. *NPJ Vaccines.* 2022;7:138. 10.1038/S41541-022-00554-1 36344523 PMC9640704

[62] Knight GM Griffiths UK Sumner T : Impact and cost-effectiveness of new tuberculosis vaccines in low- and middle-income countries. *Proc. Natl. Acad. Sci. USA.* 2014;111:15520–15525. 10.1073/pnas.1404386111 25288770 PMC4217399

[63] Weerasuriya CK Harris RC McQuaid CF : The epidemiologic impact and cost-effectiveness of new tuberculosis vaccines on multidrug-resistant tuberculosis in India and China. *BMC Med.* 2021;19:60. 10.1186/S12916-021-01932-7 33632218 PMC7908776

[64] SMART4TB: Community Consensus on the Earlier Inclusion of Pregnant Women and Persons in TB Research. 2024.

[65] Palmer S Suarez M Frick M : Elevating the Leadership of High TB Burden G20 States in the Development and Delivery of New TB Vaccines. 2024.

[66] CEPI: CEPI officially launched. 2017. (accessed March 19, 2025). Reference Source

[67] AMR Action Fund: The AMR Action Fund announces its first non-industry investments, raising an additional US$140 million toward addressing antimicrobial resistance (AMR). 2021. (accessed March 19, 2025). Reference Source

[68] UNAIDS: Domestic revenues, debt relief and development aid: Transformative pathways for ending AIDS by 2030 Report on Eastern and Southern Africa, 2024. 2024.

[69] UNAIDS: Pandemic triad: HIV, COVID-19 and debt in developing countries. 2022. 10.2989/16085906.2022.2104168 35901305

